# Prediction of visual field defects from macular optical coherence tomography in glaucoma using cluster analysis

**DOI:** 10.1111/opo.12997

**Published:** 2022-05-22

**Authors:** Janelle Tong, David Alonso-Caneiro, Michael Kalloniatis, Barbara Zangerl

**Affiliations:** 1 Centre for Eye Health, University of New South Wales (UNSW), Sydney, New South Wales Australia; 2 School of Optometry and Vision Science, University of New South Wales (UNSW), Sydney, New South Wales Australia; 3 Contact Lens and Visual Optics Laboratory, Centre for Vision and Eye Research, School of Optometry and Vision Science, Queensland University of Technology, Kelvin Grove, Queensland Australia; 4 Coronary Care Unit, Royal Prince Alfred Hospital, Sydney, New South Wales Australia

**Keywords:** glaucoma, optical coherence tomography, structure–function relationships, visual fields

## Abstract

**Purpose:**

To assess the accuracy of cluster analysis-based models in predicting visual field (VF) defects from macular ganglion cell-inner plexiform layer (GCIPL) measurements in glaucomatous and healthy cohorts.

**Methods:**

GCIPL measurements were extracted from posterior pole optical coherence tomography (OCT), from locations corresponding to central VF test grids. Models incorporating cluster analysis methods and corrections for age and fovea to optic disc tilt were developed from 493 healthy participants, and 5th and 1st percentile limits of GCIPL thickness were derived. These limits were compared with pointwise 5th and 1st percentile limits by calculating sensitivities and specificities in an additional 40 normal and 37 glaucomatous participants, as well as applying receiver operating characteristic (ROC) curve analyses to assess the accuracy of predicting VF results from co-localised GCIPL measurements.

**Results:**

Clustered models demonstrated globally low sensitivity, but high specificity in the glaucoma cohort (0.28–0.53 and 0.77–0.91, respectively), and high specificity in the healthy cohort (0.91–0.98). Clustered models showed similar sensitivities and superior specificities compared with pointwise methods (0.41–0.65 and 0.71–0.98, respectively). There were significant differences in accuracy between clusters, with relatively poor accuracy at peripheral macular locations (*p* < 0.0001 for all comparisons).

**Conclusions:**

Cluster analysis-based models incorporating age correction and holistic consideration of fovea to optic disc tilt demonstrated superior performance in predicting VF results to pointwise methods in both glaucomatous and healthy eyes. However, relatively low sensitivity and poorer performance at the peripheral macula indicate that OCT in isolation may be insufficient to predict visual function across the macula accurately. With modifications to criteria for abnormality, the concepts suggested by the described normative models may guide prioritisation of VF assessment requirements, with the potential to limit excessive VF testing.

**Supplementary Information:**

The online version of this article (doi:10.1111/opo.12997) contains supplementary material, which is available to authorized users.

## Key points

- Compensation for age, eccentricity and fovea to optic disc tilt-related variations in ganglion cell-inner plexiform layer thickness results in more accurate identification of locations corresponding to normal visual field sensitivity in healthy and glaucomatous eyes.
- Poorer performance at the peripheral compared with the central macula suggests that optical coherence tomography may be unable to predict visual field sensitivity in isolation, and may contribute to reduced concordance between reduced ganglion cell-inner plexiform layer thickness and visual field sensitivity.
- The concepts described may be useful as screening tools to aid clinical decision making, although location-specific criteria for macular abnormalities may be required to improve performance.

## INTRODUCTION

In the context of glaucoma, visual field (VF) assessment remains the clinical standard for disease staging and determining outcome measures in clinical trials,^1–3^ and provides important information on visual morbidity associated with glaucoma.^4,5^ However, despite ongoing improvements to limit the effects of extraneous factors on test performance^6,7^ due to inherent subjectivity, VF results can be notoriously variable, with high inter- and intra-individual variation in both normal and diseased populations.^8–11^ Repeated testing is often required for confirmation of VF results,^12^ which can unnecessarily delay initiation of appropriate management strategies and increase patient burden and associated costs.^13–15^ Due to these limitations, there has been increasing interest in investigating the relationship between more objective structural measures and VF sensitivity to potentially enable prediction of VF sensitivity from structure,^16–19^ which may aid targeted identification of patients who would benefit most from VF testing and provide a surrogate understanding of visual function for those patients unable to perform VF testing. Additionally, investigations of the structure–function relationship endeavour to improve identification of structure–function concordance. From a clinical perspective, concordant structural and functional findings are likely to improve discrimination between patients with glaucoma and variations of normal,^20^ and identification of progressive glaucoma requiring treatment escalation. Optical coherence tomography (OCT) remains a popular imaging modality due to its high-resolution in vivo imaging capability, rapid acquisition time and highly repeatable quantitative information.^21,22^ Proposed models predicting VF results from OCT demonstrate variable performance, in part related to variable OCT parameters, VF parameters and cohorts along different stages of the glaucoma spectrum being investigated. However, methods predicting global VF metrics have generally outperformed those attempting to predict location-specific VF thresholds from OCT data.^17,19,23^ Qualitative topographic comparisons between OCT-derived retinal nerve fibre layer (RNFL) and ganglion cell-inner plexiform layer (GCIPL) thicknesses and VF thresholds have reported global agreement between abnormal structure and function in up to 88.7% of patients with early glaucoma^17,23^ Moreover, various deep learning methods have been applied to RNFL and GCIPL data to enable prediction of summary VF metrics such as mean deviation and VF index, with moderate to strong correlations reported between predicted and actual VF parameters.^24–26^ These indicate promising utility of these methods in predicting global functional results from OCT.

While techniques obtaining global agreement between structure and function greatly limit diagnostic ambiguity, a location-specific approach is still valuable to identify areas likely to demonstrate corresponding functional deficits, in turn facilitating clinical decision-making regarding whether additional functional assessment is warranted for confirmation. However, marked inter-individual heterogeneity in various anatomical features, which are often averaged to obtain less variable global metrics, likely contributes to poorer concordance in location-specific comparisons between structure and function. For example, RNFL trajectory is influenced by inter-individual variations in the position of the optic disc relative to the fovea and optic disc insertion,^27–29^ related to refractive error to some extent,^30^ precluding a one-size-fits-all strategy of direct superimposition of structural and functional data when using the RNFL as the structural component.

Due to issues with variability inherent with RNFL analyses and pointwise methods in general, we have recently applied hierarchical and k-means cluster algorithms, which are various types of cluster analysis methods, to group retinal thickness data based on specified statistical criteria. These methods enabled identification of locations within the macular ganglion cell layer (GCL) showing statistically similar ageing properties,^31^ and the resultant redistribution of inter- and intra-individual variability in otherwise highly variable data has enabled robust identification of location-specific changes in the GCL in age-related macular degeneration.^32^ Moreover, quantitative prediction of VF results from OCT data is hampered by high variability in both VF and OCT findings within glaucoma cohorts,^19,33,34^ which may be overcome by focusing on binarised classifications of VF and OCT data falling within or outside of normative limits. Applying these approaches in glaucoma may be able to bypass inherent inter-individual structural variability, in turn improving predictability of defective VF locations from OCT.

In the present study, we assessed the accuracy of cluster analysis-based methods, developed from a representative normative cohort, in identifying corresponding qualitative VF loss from OCT-derived macular GCIPL thickness in glaucomatous and healthy cohorts, and compared that with the conventional pointwise approach. We hypothesise that methods applying unsupervised cluster analysis, where data are automatically segregated into groups with similar characteristics per operator-specified separability criteria, provide a robust and less variable normative comparison, thereby improving identification of defective locations in the VF from OCT data. The described techniques have the potential to form the basis of clinical implementations to aid early diagnosis of glaucoma.

## METHODS

### Study cohorts

The study cohort consisted of 493 retrospectively recruited healthy participants (hereby the normative model cohort), 37 prospectively recruited glaucoma participants and 40 prospectively recruited healthy participants (hereby the healthy testing cohort, to distinguish from the normative model cohort) from the Centre for Eye Health (Sydney, Australia). All participants underwent at least one comprehensive eye examination including slit-lamp biomicroscopy, intraocular pressure measurement, dilated fundus examination, OCT imaging of the macula and peripapillary RNFL (Cirrus OCT, Carl Zeiss Meditec, zeiss.com/meditec and Spectralis OCT, Heidelberg Engineering, heidelbergengineering.com) and 24–2 Swedish Interactive Threshold Algorithm (SITA) Standard or SITA Faster threshold VF testing (Humphrey field analyser (HFA), Carl Zeiss Meditec, zeiss.com/meditec). Healthy participants were identified from this examination as those with no optic nerve pathology in either eye. All glaucoma participants had received their diagnosis from a glaucoma specialist ophthalmologist, and either previously underwent selective laser trabeculoplasty or peripheral iridotomy or were prescribed topical therapy at the time of recruitment. The majority of participants were classified as having early glaucoma, as described in previous studies.^35,36^ Data from all of the healthy participants and 25 of the glaucoma participants have been reported in part in previous investigations.^31–33,36–39^ As per previous studies,^31–33,37^ exclusion criteria for all cohorts included spherical equivalent refractive error outside the range of ±6.00 dioptres (D), astigmatism greater than 3.00 D and the presence of macula pathology in the included eye. Written consent to utilise clinical data for research purposes was obtained as per protocols approved by the University of New South Wales, Australia, Human Research Ethics Advisory Panel. This study adhered to the tenets of the Declaration of Helsinki.

### Development of cluster analysis-based normative models

**FIGURE 1:**
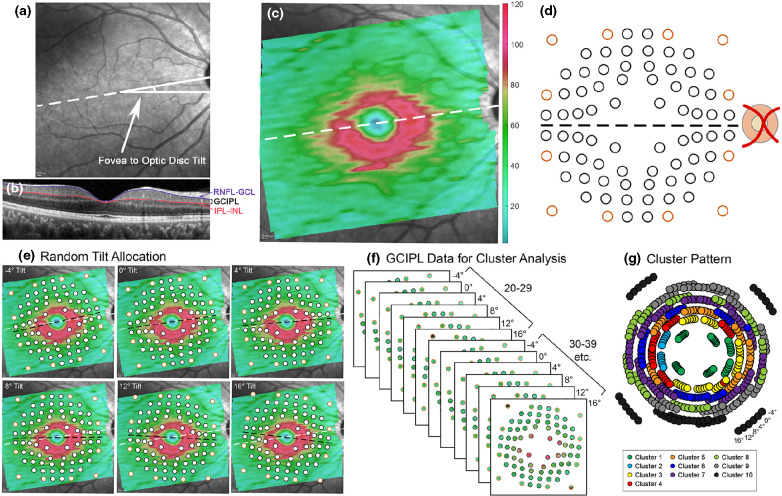
Schematic describing ganglion cell-inner plexiform layer (GCIPL) measurement extraction from the normative model cohort for normative model development. (a) The infrared posterior pole image with the fovea to optic disc tilt (the angle between the foveal and optic disc centres) labelled with the white dashed line. (b) The central B-scan, coinciding with the fovea to optic disc tilt labelled in (a), showing the retinal nerve fibre layer (RNFL)-ganglion cell layer (GCL) boundary and inner plexiform layer (IPL)-inner nuclear layer (INL) boundary between which GCIPL measurements were extracted. (c) GCIPL thickness map generated by the MATLAB algorithm. The colour bar indicates GCIPL thickness measurements in microns. (d) Humphrey field analyser (HFA) 10–2 test locations (black) and paracentral 30–2 test locations (orange) displaced using Drasdo correction, 43 as visible by the variable spacing between test locations, indicating the areas over which GCIPL measurements were extracted by the MATLAB algorithm. Grid tilt is delineated by the black dashed line. (e) The normative model cohort was randomly assigned a fovea to optic disc tilt of −4°, 0°, 4°, 8°, 12° or 16°, and the tilt of the extraction grid was adjusted based on the allocated tilt relative to individual participants' fovea to optic disc tilt. (f) Hierarchical cluster analysis was applied to GCIPL data grouped by decade bracket (20–29 years, 30–39 years etc. up to 70–84 years) and assigned fovea to optic disc tilt. (g) A cluster pattern derived from F, where locations of the same colour indicate those that show statistically similar ageing properties. At each measurement location, the six spots rotating clockwise represent measurements extracted at −4° to 16° fovea to optic disc tilts, separated by 4° intervals.

To address previously identified factors contributing to normal inter-individual variability, data from the normative model cohort were used to develop normative models of the macular GCIPL, and the ability of these models to identify VF-defective locations was tested in subsequent analyses. Macular OCTs were acquired using the Spectralis OCT posterior pole volume scan setting, spanning 30° horizontally and 25° vertically, and centred on the foveal pit (Figure 1). Where one eye was eligible for inclusion, OCT from this eye was included for further analysis, while if both eyes were eligible, one eye was selected at random for inclusion. Automated segmentation of the GCL and IPL boundary positions was manually reviewed and corrected, with locations that could not be accurately segmented due to anatomical artefacts such as blood vessel shadowing excluded from further analyses. Details of individual processes of model development can be found in the Appendix S1; however, the main components are described herein.

**FIGURE 2:**
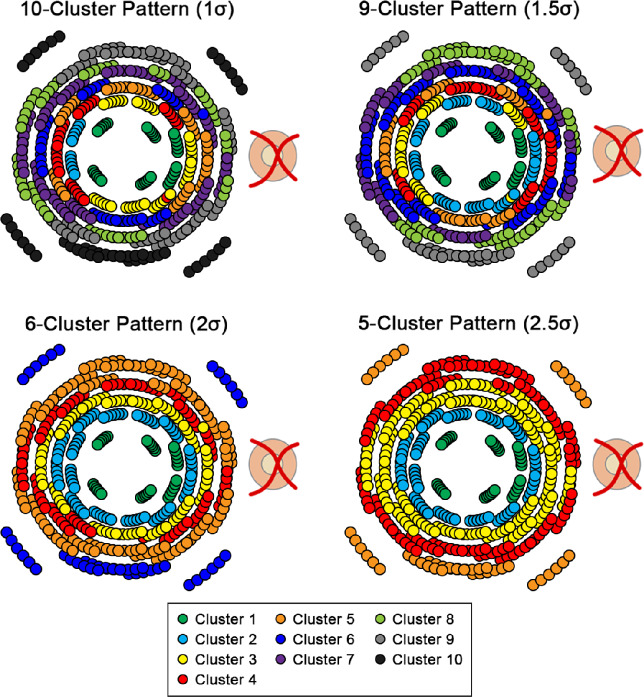
All cluster patterns derived from hierarchical cluster analysis methods applied to ganglion cell-inner plexiform layer (GCIPL) thickness measurements using the various d’ criteria to determine cluster separability, as described in detail in the Supplementary Methods in Appendix S1. Within each pattern, locations of the same colour indicate those showing statistically similar ageing properties.

In the light of a large spread of population fovea to optic disc tilts and its contribution to inter-individual variations in the structure–function relationship,^40^ the normative model cohort was randomly allocated to a fovea to optic disc tilt of −4°, 0°, 4°, 8°, 12° or 16°. This was performed to evenly distribute participants over the range of likely fovea to optic disc tilts, rather than using individuals' fovea to optic disc tilts, which would result in few participants at the extreme ends of this range. Macular GCIPL thicknesses were extracted over locations corresponding to the HFA 10–2 and 12 paracentral locations from the 30–2, with correction of test target locations for lateral displacement of ganglion cells (Figure 1),^41,42^ and at the allocated fovea to optic disc tilt using a custom MATLAB (MathWorks, mathworks.com) algorithm.^39^ After data extraction, all left eye data were converted to the right eye format. As per previous studies, hierarchical cluster analysis was then applied to GCIPL data grouped by age into decade brackets (20–29 years, 30–39 years… up to 70–84 years) and allocated fovea to optic disc tilt, to identify macular GCIPL locations demonstrating similar ageing properties (Figure 1).^31,37,43^ By identifying locations that are suitable to pool together without predetermined assumptions based on data point locations, intra-individual variations in GCIPL thickness with eccentricity can be minimised. As hierarchical cluster analysis does not involve *a priori* assumptions on the number of clusters, d’ criteria of d’ = 1, 1.5, 2 and 1.5 were applied, equivalent to minimum inter-cluster separation of 1 standard deviation (σ), 1.5σ, 2σ and 2.5σ and resulting in generation of patterns with 10, 9, 6 and 5 statistically separable clusters, respectively (Figure 2). All patterns followed a quasi-concentric configuration consistent with previous work in this area^31,37^ and histological models of ganglion cell density,^44^ and were tested in subsequent analyses to investigate whether varying degrees of separation in GCIPL thickness between clusters would significantly alter model performance.

The derived cluster patterns were subsequently used to develop ageing models with best-fit quadratic or linear regression models, to be applied as age-correction factors to minimise variability due to normal ageing changes.^33^ Per previously described methods,^31^ participants in the normative model cohort were grouped into decade brackets (20–29 years, 30–39 years… to 70+ years), and mean GCIPL thickness measurements were calculated for each age bracket and cluster per cluster pattern. Extra sums-of-squares F tests were used to compare quadratic and linear regression models applied to describe GCIPL thickness as a function of age. Finally, while refractive error was considered due to previous reports of decreased inner retinal thickness with increasing myopia,^45,46^ significant relationships between refractive error and clustered GCIPL thickness were only observed consistently in the peripheral-most cluster, and were subsequently not incorporated into the normative models.

### Derivation of percentile normative limits from clustered and pointwise methods

**FIGURE 3:**
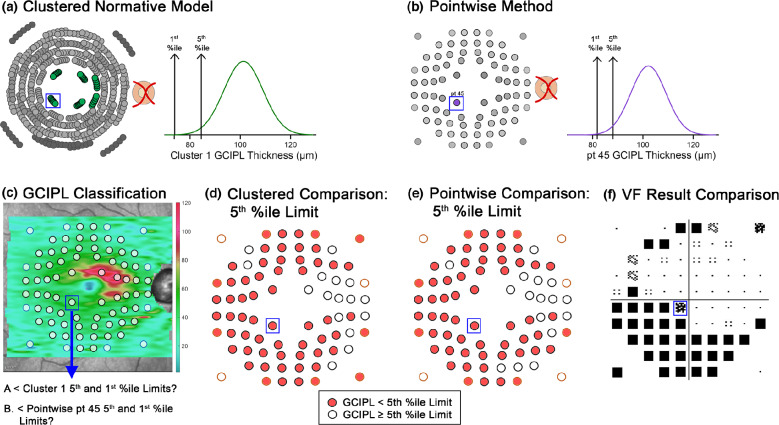
Schematic describing methods to derive ganglion cell-inner plexiform layer (GCIPL) limits, with a single central location (point [pt] 45, blue box) highlighted as a demonstrative example. (a) As pt 45 falls within Cluster 1 of the 10-cluster pattern (green circles), the 5th and 1st percentiles' (%ile) limits were obtained from the distribution of all cluster 1 locations within the normative model cohort (black arrows). (b) For pointwise methods, 5th and 1st percentile limits were derived from the individual normative distribution for pt 45. (c) A GCIPL thickness map and overlying optical coherence tomography (OCT) extraction grid (circles) for a 44-year-old participant in the glaucoma cohort. (d) The GCIPL thicknesses at each location were compared with the corresponding limits derived from 5th percentile normative limits per the clustered normative model, and subsequently classified as below or above this cut-off. For pt 45 (blue box), this was compared with the 5th percentile limit from Cluster 1 per a. (e) Similarly, the GCIPL thicknesses were compared with pointwise 5th percentile limits; for pt 45, this is the limit described in (b). For both (d and e), this process was repeated for derived 1st percentile limits. (f) GCIPL classifications were then compared with the corresponding VF location (blue box), with VF results flipped vertically to match structure.

As 5th and 1st percentile normative limits of GCIPL thickness are typically used in commercial OCT software to signify borderline and outside normative limits, respectively, these limits were derived for the clustered normative models and more conventional pointwise methods to enable binarisation of data to within or outside normative limits. For the clustered normative models, GCIPL thicknesses from the normative model cohort were pooled by cluster and converted to 50-year-old equivalents using cluster-specific age-correction factors derived from clustered regression models (Table S3), and the 5th and 1st percentile normative limits were calculated per cluster. After age correction of the glaucoma and healthy testing cohorts, GCIPL thicknesses for each individual location (i.e., not pooled) were compared with the 5th and 1st percentile normative limits for the cluster in which the particular location fell (Figure 3), and were subsequently flagged as falling within or outside of these limits. Comparison with a cluster of normative data points, as identified by the normative models, produces percentile limits that are less likely to be impacted by eccentricity-related variability.^37^

Meanwhile, for pointwise analyses, the 5th and 1st percentile normative limits were calculated at each location without age correction from normative model cohort data, with GCIPL thicknesses re-extracted at 0° grid tilt irrespective of fovea to optic disc tilt (Figure 3). This was conducted to more closely resemble analyses performed by conventional OCT review software, while incorporating findings from a previous study demonstrating significantly different GCIPL values when extracting over areas matching central VF test paradigms.^39^ Similar to clustered analyses, GCIPL thickness measurements across the macula from both glaucoma and healthy testing cohorts were compared with the corresponding 5th and 1st percentile normative limits, and locations were flagged as falling within or outside of the derived limits.

### Testing clustered and pointwise methods on glaucoma and healthy data

For the glaucoma and healthy testing groups, on which the clustered normative models and more conventional pointwise methods were compared, macular OCTs were acquired and extracted using an identical protocol to the normative modelling group, with the exception of grid tilt set at 0° regardless of individual fovea to optic disc tilts, to reflect the retinal locations stimulated during VF testing in a natural head position. These cohorts also underwent additional HFA testing on the 10–2 and 30–2 test grids with Goldmann III (GIII) stimulus sizes and full-threshold strategies for the eye included in structural analyses, as per protocols described in previous studies,^31,33^ with full threshold chosen due to its increased consistency regardless of participant experience with VF testing.^47^ Each test grid was repeated once, totalling four VFs per participant. VFs were performed in random order, and rest breaks were offered to minimise systematic order and fatigue effects. VFs exceeding manufacturer-specified criteria of 20% fixation losses and 15% false positives, in conjunction with gaze tracker information, were considered unreliable and excluded. From the 10–2 and 12 paracentral locations from the 30–2 converted to right eye format, individual locations were assigned as VF-defective if the pattern deviation *p* values met the following criteria:

- *p* < 2%, repeatable across both VF tests;
- *p* < 1% or *p* < 0.5%, with at least *p* < 5% on repeat VF testing.

All other locations were assigned as VF normal, to enable binarisation of VF data for comparison with GCIPL classifications (Figure 3).

Statistical analyses were performed using GraphPad Prism version 8.4.3 (GraphPad, graphpad.com). Normality was assessed using D'Agostino and Pearson’s tests, with appropriate test types chosen based on normality results. Differences in demographic variables between the normative model, healthy testing and glaucoma cohorts were compared using Kruskal–Wallis tests with Dunn's multiple comparison tests for continuous variables and chi-squared tests for categorical variables.

To compare the ability of the clustered normative models and pointwise methods in identifying the presence of global structure–function concordance within the glaucoma cohort, for the derived 5th and 1st percentile limits, individual test locations were classified as follows:

- true positive (TP): GCIPL flagged as below cut-off and VF-defective;
- false positive (FP): GCIPL flagged as below cut-off and VF-normal;
- false negative (FN): GCIPL not flagged and VF-defective;
- true negative (TN): GCIPL not flagged and VF-normal.

As per Hood et al.,^23^ the number of participants within the glaucoma cohort with at least two contiguous TP locations was identified to determine the prevalence of abnormal structure–abnormal function concordance within this group. Similarly, the presence of at least two contiguous FP locations was used to determine the prevalence of abnormal structure–normal function.

Overall and cluster-specific sensitivities and specificities for each clustered normative model were derived using the cluster-based 5th and 1st percentile limits, and global sensitivity and specificity values were compared with those derived from pointwise methods. These analyses were conducted for both the glaucomatous and healthy testing cohort. As the healthy testing cohort did not demonstrate any notable VF defects, locations were classified as FP or TN as per the above. Ninety-five per cent (95%) confidence intervals for each parameter were calculated as described by Ying et al.^48^ to aid comparisons between methods. This calculation requires the total prevalence of VF defects across all VF locations in the glaucoma cohort, calculated at 9.79% (Figure S1).

To aid visualisation of cluster-specific performance for each normative model, within the glaucoma cohort individual GCIPL locations flagged as TP and FN were pooled as VF-defective, while FP and TN locations were pooled as VF-normal. For each clustered normative model, GCIPL measurements were subsequently pooled by cluster to generate cluster-specific receiver operating characteristic (ROC) curves. These analyses were performed for the glaucoma cohort only, to avoid inflation of model specificity by the inclusion of healthy testing cohort data. Areas under the ROC curves (AUROCs) were derived and compared between clusters within individual cluster patterns using Welch's analysis of variance (ANOVA) with Dunnett's T3 multiple comparisons tests.

To determine whether 5th and 1st percentile normative limits were optimal in identifying VF defects based on GCIPL thicknesses, from ROC analyses cut-off GCIPL values were chosen using the maximum Youden's index, where the sum of the sensitivity and specificity values is at their maximum.^49^ Percentile ranks of cluster-based cut-off values derived from ROC analyses relative to the normative model cohort were calculated as points of comparison with those derived from percentile normative limits. ROC-generated GCIPL cut-offs were then reapplied to the glaucoma and healthy testing cohort as per percentile limits to calculate sensitivity and specificity, with the caveat that this may overestimate model performance in the glaucoma cohort due to resampling.^50^ Throughout this study, the level of statistical significance was set at *p* < 0.05. 

## RESULTS

### Demographic characteristics

Table 1 lists the demographic characteristics of the two healthy cohorts (normative model and healthy testing) and the glaucoma cohort. The glaucoma cohort was significantly older and more hyperopic than the healthy cohorts (*p* < 0.0001 and 0.02, respectively, Kruskal–Wallis test), and the fovea to optic disc tilts were significantly smaller in the healthy testing cohort (*p* = 0.008, Kruskal–Wallis test). As expected, the mean and pattern standard deviations were also significantly worse in the glaucoma cohort (*p* < 0.0001 for both, Kruskal–Wallis test). No other variables differed significantly between cohorts.

**TABLE 1:** Demographic characteristics of the included cohorts

	*N*	Age (y)^a^	Spherical equivalent (D)^a^	Eye (OD:OS)^b^	Gender (M:F)^b^	Fovea to optic disc tilt (°)^a^	IOP (mmHg)^a^	VF MD (dB)^a^	VF PSD (dB)^a^
Normative model cohort	493	47.41 ± 16.02	−0.56 ± 1.82	257:236	213:280	6.83 ± 3.33	15.78 ± 3.02	−0.57 ± 1.85	1.79 ± 1.17
Healthy testing cohort	40	46.00 ± 14.49	−0.90 ± 2.13	24:16	19:21	5.19 ± 3.79	15.55 ± 2.91	0.45 ± 0.95	1.42 ± 0.28
Glaucoma cohort	37	64.71 ± 7.44	0.03 ± 2.63	18:19	22:15	7.38 ± 3.81	15.05 ± 3.00	−2.11 ± 2.13	3.53 ± 2.33
*p* value		<0.0001	0.02	0.80	0.15	0.008	0.42	<0.0001	<0.0001

*Note:* The normative model cohort was used to develop the clustered normative models and derive 5th and 1st percentile limits using both clustered and pointwise methods, which were subsequently tested on the healthy testing and glaucoma cohorts. Where applicable, all values indicate the mean ± standard deviation.

Abbreviations: °, degrees; D, dioptres; dB, decibels; F, female; IOP, intraocular pressure; M, male; MD, mean deviation; mmHg, millimetres of mercury; *N*, number of participants; OD, right eye; OS, left eye; PSD, pattern standard deviation; VF, visual field; y, years of age.

^a^ Kruskal–Wallis test with multiple comparisons.

^b^ Chi-squared test.

### Global assessments of structure–function concordance

Firstly, global measures of abnormal structure–abnormal function concordance in the glaucoma cohort were calculated using 5th and 1st percentile limits derived from pointwise methods and the clustered normative models, to serve as a point of comparison with previous work.^23^ From the 19 participants in the glaucoma cohort (51%) with VF defects within the 10–2 and/or paracentral 30–2 locations, 12 to 17 participants (63.2–84.2%) demonstrated abnormal structure–abnormal function concordance when applying 5th percentile limits derived from the various clustered normative models (Table 2). These were superior to 1st percentile limits across all methods, where only 8–10 participants (42.1–52.6%) demonstrated abnormal structure–abnormal function concordance. While the models with a greater number of clusters (namely the 10-cluster and 9-cluster patterns, with minimum 1 and 1.5σ between clusters, respectively) showed comparable performance to pointwise methods, the models with fewer clusters (and therefore greater σ separability between clusters) were poorer overall at identifying abnormal structure–abnormal function concordance. However, the 18 glaucoma participants without VF defects all showed abnormal GCIPL thicknesses regardless of whether 5th or 1st percentile limits from pointwise or clustered normative model methods were applied, indicating a notable proportion of abnormal structure–normal function in the present glaucoma cohort.

**TABLE 2:** Number and percentage of participants within the glaucoma cohort demonstrating abnormal structure–abnormal function concordance in at least two contiguous locations, using different ganglion cell-inner plexiform layer (GCIPL) criteria and clustered normative models

	5th percentile limits	1st percentile limits
Pointwise	17 (89.5%)	12 (63.2%)
10-cluster pattern (1σ)	16 (84.2%)	10 (52.6%)
9-cluster pattern (1.5σ)	16 (84.2%)	10 (52.6%)
6-cluster pattern (2σ)	16 (84.2%)	8 (42.1%)
5-cluster pattern (2.5σ)	12 (63.2%)	8 (42.1%)

*Note:* Percentages were calculated as a proportion of the glaucoma cohort demonstrating visual field (VF) defects (*N* = 19).

Abbreviation: σ, standard deviations.

### Sensitivities and specificities of clustered and pointwise methods

Location-specific performance of the clustered normative models was subsequently assessed against pointwise methods, by comparing the ability of the derived 5th and 1st percentile limits to correctly identify corresponding VF defects across the glaucoma cohort and the absence of VF defects in the healthy cohort. Within the glaucoma cohort, specificities derived from clustered normative models were moderate to high, and were significantly better than those obtained with pointwise methods (Table 3). That is, the models were more likely to correctly identify GCIPL thicknesses corresponding to VF-normal locations in glaucoma participants, with a maximum increase in correct identification of VF-normal locations of 9%. Furthermore, within the healthy cohort, specificities were comparable between pointwise methods and the various clustered normative models, and were relatively high across all methods. Overall, specificities were higher in the healthy testing cohort relative to the glaucoma cohort, and when applying 1st percentile limits compared with 5th percentile limits.

**TABLE 3:** Accuracy of 5th and 1st percentile normative limit ganglion cell-inner plexiform layer (GCIPL) cut-offs, derived from the normative model cohort, in identifying co-localised visual field (VF)-defective and VF-normal results in the glaucoma and healthy testing cohorts

	Glaucoma		Healthy	
	Sensitivity	Specificity	Specificity	
5th percentile limits				
Pointwise	0.65 (0.59–0.71)	0.71 (0.69–0.72)	0.92 (0.91–0.93)	
10-cluster pattern (1σ)	0.53 (0.47–0.59)	0.77 (0.76–0.79)	0.92 (0.91–0.93)	
9-cluster pattern (1.5σ)	0.54 (0.48–0.60)	0.77 (0.76–0.79)	0.92 (0.91–0.93)	
6-cluster pattern (2σ)	0.51 (0.45–0.57)	0.78 (0.77–0.80)	0.91 (0.90–0.92)	
5-cluster pattern (2.5σ)	0.49 (0.43–0.55)	0.80 (0.79–0.82)	0.92 (0.91–0.93)	
1st percentile limits				
Pointwise	0.41 (0.35–0.47)	0.86 (0.85–0.87)	0.98 (0.98–0.99)	
10-cluster pattern (1σ)	0.31 (0.26–0.37)	0.90 (0.88–0.91)	0.98 (0.97–0.98)	
9-cluster pattern (1.5σ)	0.31 (0.26–0.37)	0.90 (0.89–0.91)	0.98 (0.97–0.98)	
6-cluster pattern (2σ)	0.28 (0.22–0.33)	0.90 (0.89–0.91)	0.98 (0.98–0.99)	
5-cluster pattern (2.5σ)	0.28 (0.23–0.34)	0.91 (0.90–0.92)	0.98 (0.98–0.99)	

*Note:* 95% confidence intervals are shown in brackets. Details of cluster-specific accuracies can be found in Table S1.

Abbreviation: σ, standard deviations.

Meanwhile, sensitivities derived from the clustered normative models were low, particularly compared with the specificity outputs, indicating poor overall ability to correctly identify GCIPL thicknesses corresponding to VF-defective locations. While no significant differences in sensitivity between the clustered normative models and pointwise methods were suggested by overlapping 95% confidence intervals, numerically sensitivity values using the clustered normative models were poorer, with a maximum decrease in correct identification of VF-defective locations of 16%. Poorer sensitivities were observed with 1st percentile limits relative to 5th percentile limits, highlighting the trade-off in sensitivity, which may be associated with high specificity. Comparisons between the different clustered normative models did not reveal significant differences between sensitivity and specificity outputs; however, a trend of decreasing sensitivity and increasing specificity was observed with decreasing number of clusters. For location-specific variations in pointwise methods, specificities across the macula were reasonably consistent within the healthy testing cohort. However, within the glaucoma cohort sensitivities and specificities varied widely, ranging between 0 and 1 and between 0.37 and 0.97, respectively, using 5th percentile limits (Figure S2).

**FIGURE 4:**
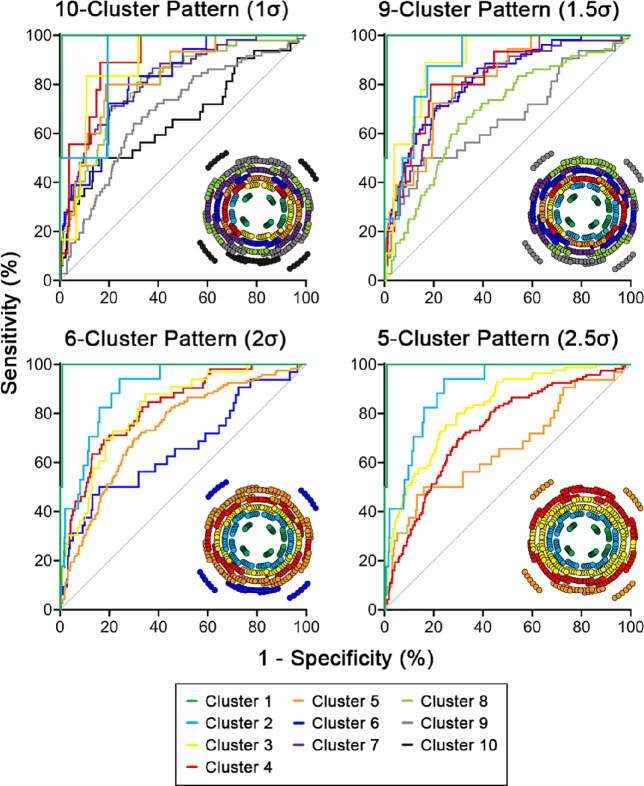
Receiver operating characteristic (ROC) curves per cluster within each cluster pattern, describing variations in sensitivity and specificity of ganglion cell-inner plexiform layer (GCIPL) thickness cut-offs to identify visual field (VF)-normal and VF-defective locations. Analyses were performed for the glaucoma cohort only, to avoid inflation of model specificity. The diagonal line is the no-discrimination line, where classification into VF-normal and VF-defective would occur at random. Corresponding cluster patterns from Figure 2 are included as insets for clarity.

Within cluster patterns, inter-cluster comparisons revealed that sensitivities tended to be highest at the central clusters and deteriorated notably with increased eccentricity, while specificities were generally consistent across clusters (Table S1). Additionally, within the glaucoma cohort, ROC curves generated for individual clusters for each clustered normative model demonstrated high model accuracy for the central to mid-peripheral clusters, with AUROCs of at least 0.80 indicating excellent diagnostic accuracy.^51,52^ However, poorer accuracy at peripheral macular locations was observed, where curves approached the no-discrimination line and AUROCs ranged between 0.66 and 0.74 across all models (Figure 4 and Table 4). Cluster-specific differences in model accuracy were also reflected in significantly lower AUROCs in peripheral versus central clusters (*p* < 0.0001 for all comparisons, Welch's ANOVA; Table 4).

**TABLE 4:** Areas under the receiver operating characteristic curves (AUROCs) for ganglion cell-inner plexiform layer (GCIPL) thicknesses matching visual field (VF)-defective locations in the glaucoma cohort, with 95% confidence intervals in brackets

	10-cluster pattern (1σ)	9-cluster pattern (1.5σ)	6-cluster pattern (2σ)	5-cluster pattern (2.5σ)
Cluster 1	1.00 (0.99–1.00)	1.00 (0.99–1.00)	1.00 (0.99–1.00)^a^	1.00 (0.99–1.00)^a^
Cluster 2	0.90 (0.76–1.00)	0.89 (0.82–0.96)^a^	0.90 (0.84–0.95)^a^	0.90 (0.84–0.95)^a^
Cluster 3	0.89 (0.80–0.97)	0.90 (0.83–0.97)	0.82 (0.75–0.88)	0.82 (0.78–0.87)^a^
Cluster 4	0.90 (0.83–0.97)	0.83 (0.74–0.93)	0.82 (0.77–0.88)^a^	0.74 (0.69–0.78)^a^
Cluster 5	0.83 (0.74–0.93)	0.82 (0.73–0.90)	0.74 (0.69–0.78)^a^	0.66 (0.55–0.77)
Cluster 6	0.82 (0.73–0.90)	0.82 (0.76–0.88)	0.66 (0.55–0.77)	
Cluster 7	0.82 (0.76–0.88)	0.80 (0.74–0.87)		
Cluster 8	0.80 (0.74–0.87)	0.69 (0.62–0.75)^a^		
Cluster 9	0.69 (0.62–0.75)	0.66 (0.55–0.77)		
Cluster 10	0.66 (0.55–0.77)			

*Note:* Within cluster patterns, comparisons between clusters showed significant differences in AUROCs (*p* < 0.0001 for all, Welch's analysis of variance).

Abbreviation: *σ,* standard deviations.

^a^ Clusters that showed significant differences in AUROCs when compared to each other within the corresponding cluster pattern (*p* < 0.05, Dunnett's T3 multiple comparisons test).

### Comparison of receiver operating characteristic (ROC) curve-based versus percentile ganglion cell-inner plexiform layer (GCIPL) cut-offs

Lastly, we sought to identify whether the relatively low sensitivities obtained with the clustered normative models, particularly at peripheral macular locations, were artefacts of the choice of GCIPL thickness cut-offs rather than reflective of poor model performance as a whole. When compared to cluster-based normative distributions within the healthy cohort, ROC cut-offs based on maximum Youden's indices fell well within the 1st percentile for the central and paracentral clusters (Table 5). Meanwhile, for peripheral macular clusters, percentile rankings varied greatly depending on the cluster pattern; however, they reached up to the 16th percentile of the normative model cohort's distribution.

**TABLE 5:** Ganglion cell-inner plexiform layer (GCIPL) thickness cut-offs per cluster derived from the maximum Youden's indices (maximum combined sensitivity and specificity) from receiver operating characteristic (ROC) curve analyses in the glaucoma cohort, using the clustered normative models

	Youden		5th percentile limit (μm)	1st percentile limit (μm)
	Cut-off (μm)	Percentile rank		
10-cluster pattern (1σ)				
Cluster 1	53.6	0.08	84.9	73.7
Cluster 2	63.6	0.07	78.0	72.0
Cluster 3	61.2	0.45	71.1	63.6
Cluster 4	59.3	0.67	66.6	61.0
Cluster 5	58.9	3.97	60.0	54.5
Cluster 6	54.6	4.50	55.2	50.5
Cluster 7	47.8	3.11	49.1	44.7
Cluster 8	44.5	5.79	44.1	40.0
Cluster 9	43.7	15.44	40.1	36.0
Cluster 10	35.6	16.72	31.2	26.8
9-cluster pattern (1.5σ)				
Cluster 1	53.5	0.08	84.8	73.7
Cluster 2	63.6	0.60	72.2	64.8
Cluster 3	59.2	0.66	66.5	61.0
Cluster 4	58.8	3.86	59.9	54.5
Cluster 5	54.5	4.42	55.2	50.4
Cluster 6	47.8	3.11	49.1	44.7
Cluster 7	44.5	5.80	44.1	40.0
Cluster 8	43.7	15.44	40.1	36.0
Cluster 9	35.6	16.72	31.2	26.8
6-cluster pattern (2σ)				
Cluster 1	53.3	0.08	84.9	73.8
Cluster 2	65.6	2.52	69.1	62.4
Cluster 3	59.2	8.56	56.9	51.8
Cluster 4	49.0	3.29	50.2	45.8
Cluster 5	44.5	12.94	41.3	37.1
Cluster 6	35.4	15.85	31.4	26.9
5-cluster pattern (2.5σ)				
Cluster 1	53.5	0.08	84.8	73.8
Cluster 2	65.7	2.60	69.1	62.5
Cluster 3	54.5	9.18	52.0	47.6
Cluster 4	44.5	12.94	41.3	37.1
Cluster 5	35.4	15.85	31.4	26.9

*Note:* Percentile ranks were calculated relative to the normative model cohort. The 5th and 1st percentile limits, also derived from the normative model cohort, are shown as points of comparison.

Abbreviations: μm, micrometres; σ, standard deviations.

**FIGURE 5:**
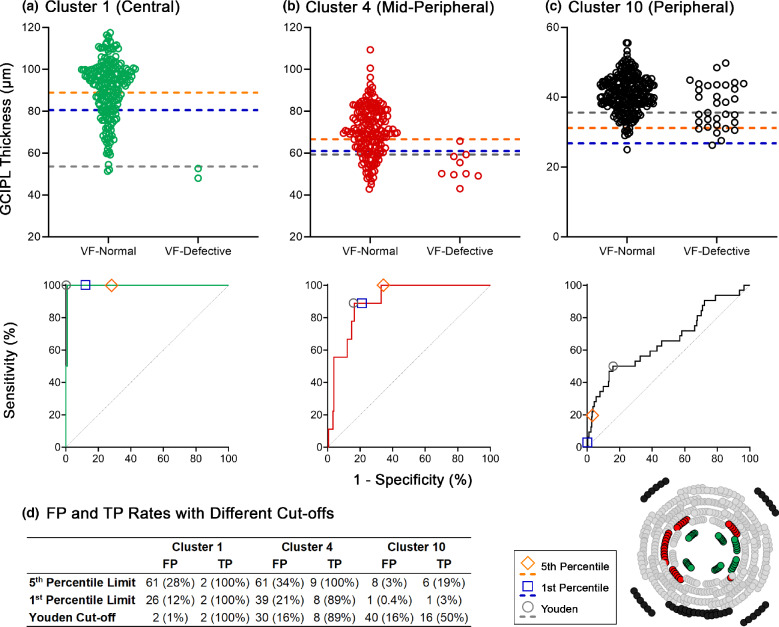
Comparison of false-positive (FP) and true-positive (TP) rates within the glaucoma cohort for different ganglion cell-inner plexiform layer (GCIPL) thickness cut-offs for central, mid-peripheral and peripheral clusters in the 10-cluster pattern (Figure 2 and bottom-right), illustrated with scatter plots and receiver operating characteristic (ROC) curves. These analyses were not performed in the healthy testing cohort, to avoid inflation of specificity. For the scatter plots, FP and TP are indicated by points falling below the dotted lines for the visual field (VF)-normal and VF-defective distributions, respectively, that is GCIPL thicknesses that would be flagged outside of normative limits for these distributions. (a) For Cluster 1, all cut-offs detect the two TP cases; however, the 5th and 1st percentile limits also result in a high FP rate relative to the Youden cut-off. This is reflected in the ROC curve, where the Youden cut-off corresponds to a higher combined sensitivity and specificity relative to the percentile limits. (b) For Cluster 4, the Youden and 1st percentile cut-offs are much closer together and perform similarly as a result, while the 5th percentile limit results in one extra TP detected at the cost of a higher FP rate. The 1st percentile limit is essentially at the highest combined sensitivity and specificity, shown by its proximity to the Youden cut-off on the ROC curve. (c) For Cluster 10, the 5th and 1st percentile limits demonstrate low FP rates at the cost of low TP rates. Meanwhile, the Youden cut-off demonstrates increased FP and TP rates; the poorer performance can be attributed to the large overlap in distributions of GCIPL thicknesses between the VF-normal and VF-defective locations. The ROC curve illustrates that the 5th and 1st percentile limits favour high specificity over sensitivity. (d) The FP and TP rates for (a-c).

To illustrate differences between GCIPL thickness cut-offs derived from ROC analyses and the more conventional 5th and 1st percentile limits in correct identification of VF-normal and VF-defective locations, scatter plots of GCIPL thicknesses were generated with these cut-offs superimposed (Figure 5). This was performed for clusters at various macular locations in the 10-cluster pattern, as this model demonstrated the best performance using percentile limits. For central macular locations, percentile limits accurately detected both VF-defective locations. However, they also incorrectly flagged a large proportion of VF-normal locations. The Youden cut-off, at the 0.08th percentile relative to the normative model cohort, demonstrated a much lower false-positive rate while detecting both true-positive data points. Meanwhile, with increasing eccentricity, the greater overlap in GCIPL thicknesses between VF-normal and VF-defective locations contributes to overall poor discrimination regardless of the chosen cut-off, with percentile limits favouring high specificity over sensitivity.

Sensitivity and specificity values of the clustered normative models when applying the Youden cut-offs to the glaucomatous and healthy testing cohort can be found in Tables S2 and S3 as a point of comparison to the percentile limits. While it should be noted that the reapplication of the Youden cut-offs onto the glaucoma cohort can overestimate model performance,^50^ these appeared comparable to slightly better than the best-performing methods using the 5th percentile limits; that is, sensitivities were comparable to pointwise 5th percentile limits but superior to clustered 5th percentile limits, while specificities were comparable to slightly better than clustered 5th percentile limits. Similarly, specificities calculated within the healthy testing cohort were also comparable between the Youden cut-offs and 5th percentile limits.

## DISCUSSION

By considering and compensating for normal anatomical variations in the macular GCIPL, normative models applying cluster analysis methods can identify VF-normal locations from OCT-derived GCIPL measures within the macular region with moderate accuracy in glaucomatous eyes, with improved performance at the central macula relative to the peripheral macula. Furthermore, clustered normative models produced comparable sensitivities but superior specificities relative to pointwise methods in the glaucoma cohort, and high specificities were found in the healthy cohort across all clustered normative models. This indicates that more holistically addressing sources of variability, such as fovea to optic disc tilt and age, can improve the detection of macular locations corresponding to normal VF sensitivities from OCT alone. However, while the clustered models can identify patients unlikely to demonstrate functional loss from macular OCTs, the poorer sensitivities observed in the glaucoma cohort across both pointwise and clustered methods indicate that OCT cannot be used in isolation to predict visual function. Nonetheless, with standard criteria of abnormality set at the 5th and 1st percentiles of the normative distributions, the moderate specificities returned by the clustered normative models suggest a low likelihood of false-positive results, that is, GCIPL thicknesses erroneously flagging VF-defective results. Potentially, in conjunction with variable criteria for abnormality to improve sensitivity, the proposed clustered models or similar paradigms may be useful as a screening tool, to identify likely VF-defective patients based on OCT and therefore those requiring additional VF testing to confirm the presence of central VF defects.

### Global structure–function concordance in previous studies

Compared with the abnormal structure–abnormal function concordance rate of 63.2–84.2% in our glaucoma cohort demonstrating central VF loss, studies utilising analogous approaches have identified similar levels of abnormal structure–abnormal function concordance, ranging from 77.4% using 10–2 alone^23^ to 82.2%–88.7% when combining 10–2 and 24–2 VF test grids.^17,23^ However, when considering the entire glaucoma cohort in this study, previously reported rates become much higher than those observed presently. Despite these studies including participants with early glaucoma, defined as 24–2 mean deviations better than −6.00 dB, it is likely that these cohorts demonstrated a high prevalence of central VF defects, which is supported by the same group reporting the presence of VF defects on 10–2 not suggested by apparently early glaucoma as per 24–2 mean deviation.^53^ As such, the cohort included in the present study may represent an even earlier stage in the glaucoma disease spectrum. Furthermore, rates of abnormal structure–abnormal function concordance alone may not be sufficient to characterise model performance; the absence of abnormal structure–abnormal function concordance could either imply normal structure–normal function concordance that is a TN result, or an error. As such, it is difficult to draw direct comparisons between the clustered normative models and other methods described by these studies.

### Advantages of clustered models over pointwise methods

While global indicators of structure–function concordance are highly practical for diagnostic purposes, these do not necessarily provide sufficient spatial detail to identify locations more likely to demonstrate concordance between OCT and VF results, and global parameters may also have limited utility in progression analyses where focal areas of change may occur.^54,55^ As such, several studies have utilised pointwise methods to quantify the structure–function relationship, in turn applying these to predict numerical VF thresholds. However, these have generally performed more poorly than global, probability-based analyses, with highly variable and relatively poor correspondence between actual and predicted VF results previously reported in glaucoma cohorts.^16,17,19,56,57^ Similarly in the present study, in contrast to relatively consistent specificities across clusters, pointwise specificities within the glaucoma cohort were widely variable, indicative of underlying variability in macular GCIPL thicknesses as a result of both expected anatomical variations and variable sites of macular damage in glaucoma.^58–60^

By identifying spatial locations that demonstrate statistically similar characteristics and are therefore suitable to analyse together, cluster analysis affords redistribution of measurement variability to improve trend discrimination, especially in data with high variability such as GCIPL thickness. The benefits of cluster analysis have been previously described by Yoshioka et al.,^37^ where age-regression models of GCL thickness were far more apparent than when applying pointwise methods, and has since been applied to elucidate patterns of ageing in VFs and corneal parameters^31,43,61^ and to optimise detection of pathological changes in VF and OCT results.^32,62^ Additionally, cluster analysis allowed for the holistic incorporation of fovea to optic disc tilt into our models, which has been identified previously as a source of heterogeneity in GCIPL thicknesses in healthy cohorts that is likely to influence variability in pointwise analyses.^39,63^ The relative preservation of sensitivity but improved specificity of clustered normative models, despite some loss of spatial information as a result of cluster-based pooling, highlights the benefits of cluster analysis approaches in detection of GCIPL measurements corresponding to VF results with greater accuracy than conventional methods.

### Poorer model performance with eccentricity and the ganglion cell-inner plexiform layer (GCIPL) floor effect

It is important to note that poorer model performance was observed with increasing eccentricity, as shown by peripheral ROC curves approaching the no discrimination line, associated significantly smaller AUROCs and poorer sensitivities at more peripheral clusters. Given similar distributions in peripheral GCIPL thicknesses between VF-normal and VF-defective locations in the glaucoma cohort (Figure 5), and similar variability between healthy and glaucoma cohorts (Figure S1), it is likely that GCIPL measurements at the peripheral macula approach the GCIPL measurement floor, reported to be between 38.5 and 45 μm, which is indeed reflected in the GCIPL measurements at the peripheral macula in the studied cohorts.^42,64^ Subsequently, the small variation in GCIPL thickness resulting in a limited dynamic range would affect separability of GCIPL measurements between VF-normal and VF-defective locations, as well as between healthy and glaucoma cohorts, leading to relatively poor discriminative ability of the GCIPL at these locations. As retinal thickness measurements are not directly analogous to neuronal cellular structures, the concept of the GCIPL measurement floor suggests that changes in cellular morphology and/or physiology affecting responses to VF stimuli do not appear to correspond to a detectable decrease in inner retinal thickness at increasingly eccentric locations.^65^ As such, emergent technologies that are able to capture morphological dysfunction at a greater resolution currently afforded by OCT or physiological dysfunction at the cellular level^66^ may become particularly valuable at relatively peripheral locations, where functional deficits cannot be predicted precisely with current methods.

### Blanket percentile limits demonstrate variable performance in detecting macular visual field defects

Compared with normative distributions, the percentile ranks of GCIPL cut-offs based on ROC analyses, which optimised sensitivity and specificity within the glaucoma cohort, were widely variable across the macula. This suggests that blanket percentile limits, that is the 5th and 1st percentile limits used in this study and on commercial OCT software, are not ideal to identify macular regions likely to demonstrate VF defects, highlighting this key pitfall of predicting VF results from commercially available analyses. ROC-based GCIPL cut-offs were well below the 1st percentile for central clusters, indicating that a greater reduction in GCIPL thickness would be required prior to a corresponding VF defect being observed, which likely contributed in part to the larger AUROC observed relative to peripheral macular locations (Figure 4 and Table 4). Moreover, this indicates that 5th or 1st percentile limits will demonstrate high false positives centrally; that is, when flagged on OCT a corresponding VF defect is unlikely to be observed. Likewise, ROC-based GCIPL cut-offs reached up to the 16th percentile for peripheral macular clusters, and therefore, VF defects corresponding to these regions may be observed despite GCIPL thicknesses within the normative range as defined with conventional metrics.

In particular, perhaps it is not unexpected that a high false-positive rate was observed for the most central clusters when using conventional percentile limits (highlighted in Figure 5a), as the abnormal GCIPL measurements relative to the normative databases but apparently normal VF results using GIII may be related to GIII stimuli falling outside of the critical area in these regions resulting in oversaturation.^67–72^ Using varying stimulus sizes approximating critical areas across the 30–2 test grid, Phu et al.^73^ demonstrated that glaucomatous functional loss can be detected up to 6.94 years earlier compared with the uniform use of GIII across the macula. While it is commonly believed that smaller stimulus sizes close to the critical area demonstrate higher test–retest variability,^67,74^ this is likely related to the corresponding smaller step sizes in the thresholding algorithm rather than an inherent property of responses to smaller VF stimuli,^75^ with no additional variability observed between stimulus sizes as a property of energy.^70^ Nonetheless, strategies using smaller stimulus sizes are only available with full-threshold algorithms, with the resultant extended duration of testing limiting its current clinical applicability. Whether using VF stimuli scaled to spatial summation characteristics at the macula improves prediction of VF-defective locations from OCT-derived GCIPL measurements remains to be seen, and is worthwhile exploring in future.

### Limitations

This study has several limitations based on the characteristics of the glaucoma and healthy testing cohorts, on which the normative models were tested. Firstly, the size of these cohorts was relatively small, due to the full-threshold VF protocols applied in this study, and the glaucoma cohort was generally older than the healthy testing cohort. While GCIPL age correction has likely mitigated these age differences for the OCT data, VF probability data could not be age-corrected, and age-related declines in VF sensitivity^10,43^ may translate to slightly different threshold criteria corresponding to different probability limits between these cohorts. Additionally, due to the demographic characteristics of glaucoma patients seen at the Centre for Eye Health, relatively few participants demonstrated VF defects at the central macula, for example, with only two glaucoma participants demonstrating a VF defect in Cluster 1 (Figure 5a), and there were several locations where no glaucoma participants demonstrated a VF defect. This has the potential to overinflate model performance at central macular locations. However, as concordant structural and functional glaucomatous changes are more readily apparent in more advanced disease,^19,76^ testing model performance in the presence of the relatively subtle changes per early glaucoma was the intention of this study's cohort composition. Nonetheless, with the inclusion of a larger number of participants with advanced glaucoma and central VF defects, should the theories hold that: (1) a larger reduction in GCIPL thickness is required prior to observing a VF defect at central macular locations and (2) the GCIPL measurement floor effect contributes to poorer accuracy at peripheral macular locations; then, little difference to the results of the current study would be expected. However, the alternative possibility is that with a greater number of VF-defective locations centrally, reduced model performance will be observed. While the present findings serve as proof of concept of potential applicability of clustered models, further testing on a more diverse glaucoma cohort would enable validation of the present findings, and determination of model performance with VF data derived from SITA strategies would aid investigations of potential clinical applicability.

Furthermore, the relatively small glaucoma cohort included in this study meant that GCIPL thickness cut-offs derived from ROC analyses could not be robustly tested, as these values were derived from the glaucoma cohort, and testing these values on the same cohort may overestimate performance. However, the results in this study suggest a promising balance between sensitivity and specificity with ROC-based cut-offs relative to the percentile normal limits, with a greater number of glaucoma patient categorisation to modelling and testing cohorts possible, enabling more robust comparisons of the various methods to predict VF results from GCIPL thicknesses applied in this study.

Moreover, the accuracy of the described models in cases of high refractive error was not tested, given the limited range of refractive errors included in this study. Inclusion of additional ocular parameters such as axial length into future models would greatly aid model refinement, especially in the light of reported variations in both inner retinal thickness and spatial summation characteristics with axial length.^77–79^ Finally, the manner in which the normative models were tested means that the relative contributions of each component, cluster analysis-aided pooling, age correction and tilt correction were not assessed, and it may be useful in future studies to isolate each component to determine their importance in applications of future normative comparisons.

## CONCLUSIONS

In the present study, we describe models that consider normal inter-individual variations in the macular GCIPL with ocular anatomy and age, and we assessed their accuracy in binary classification of VF results from GCIPL in glaucomatous and healthy eyes. Cluster analysis-based models demonstrated moderate global sensitivities and specificities within the glaucoma cohort, with higher specificities compared to pointwise methods, and high specificity was also found in the healthy cohort. These indicate that clustered methods are capable of identifying macular locations demonstrating normal VF sensitivity from OCT data, particularly at central macular locations. However, relatively low sensitivities overall and poorer accuracy at the peripheral macula indicate that current models to predict visual function from OCT-derived inner retinal thickness may be insufficient to holistically describe the macular structure–function relationship. In conjunction with further investigation on the potential benefit of variable criteria for abnormality at different macular locations, the concepts described in normative model development in this study may be useful to apply in future screening tools to aid clinical decision-making and limit the burden of excessive VF testing.

## Supplementary Information

**Appendix S1**
