# Supplementary material for: Prediction of visual field defects from macular optical coherence tomography in glaucoma using cluster analysis
Source: Ophthalmic Physiol Opt. 2022 May 22;42(5):948–64. doi: 10.1111/opo.12997 (PMC9544890; doi:10.1111/opo.12997)
Supplement: Supplementary file 1 — Appendix S1 [file 44402_2022_4205005_MOESM1_ESM.docx]

**Supplementary Material for: Prediction of visual field defects from macular OCTs in glaucoma using cluster analysis**

Janelle Tong^1,2^, David Alonso-Caneiro^3^, Michael Kalloniatis^1,2^, Barbara Zangerl^1,2^

1. Centre for Eye Health, University of New South Wales (UNSW), Sydney, NSW Australia

2. School of Optometry and Vision Science, UNSW, Sydney, NSW Australia

3. Queensland University of Technology, Contact Lens and Visual Optics Laboratory, Centre for Vision and Eye Research, School of Optometry and Vision Science, QLD, Australia

4. Coronary Care Unit, Royal Prince Alfred Hospital, Sydney, New South Wales

Supplementary Methods:

- Figures: 3
- Tables: 4

Supplementary Figures: 2

Supplementary Tables: 3

Corresponding author:

Dr Barbara Zangerl

School of Optometry and Vision Science, UNSW

Sydney 2052, NSW Australia

Email: b.zangerl@unsw.edu.au

**Supplementary Methods: Normative Model Development**

The following describes individual processes involved in development of the clustered normative models used to identify VF-defective and VF-normal locations based on GCIPL thicknesses. For clarity, figures and tables included in the Supplementary Methods are labelled distinctly from other Supplementary Materials.

**1. Fovea to optic disc tilt**

Participants in the normative model cohort were randomly assigned to fovea to optic disc tilts of -4°, 0°, 4°, 8°, 12° or 16°. The test grid tilt was aligned to match individual fovea to optic disc tilts, equivalent to a population fovea to optic disc tilt of 0°, and then adjusted further to match the assigned fovea to optic disc tilt. GCIPL thicknesses were then re-extracted using the MATLAB algorithm (Figure 1).^1^

**2. Cluster analysis**

Participants were grouped by age into decade brackets (20-29 years, 30-39 years… up to 70-84 years) and by allocated fovea to optic disc tilt, and mean GCIPL thicknesses were calculated per group for each extracted location. As per previously described methods,^2, 3^ unsupervised hierarchical cluster algorithms with within-groups linkage and squared Euclidean distance were applied using SPSS Statistics Version 23.0 (IBM Corporation, New York, NY, USA). Hierarchical cluster analysis was chosen over other common methods, such as k-means, as it does not require *a priori* inputs on number of desired clusters prior to analysis, and therefore preconceived assumptions on the most suitable number of clusters do not influence the generated output. From the maximum number of clusters derived from hierarchical cluster analysis, the d’ statistic was calculated for each cluster pair a and b to determine separability of the cluster distributions:

$$d^{'}=\frac{|x_{a}-x_{b}|}{\sqrt{0.5\times\left( {\sigma_{a}}^{2}+{\sigma_{b}}^{2} \right)}}$$

Where x and σ are the means and standard deviations respectively. Clusters were systematically merged until all comparisons reached d’>1, d’>1.5, d’>2 and d’>2.5, indicating a minimum of 1, 1.5, 2 and 2.5 units of standard deviation separating all cluster means within the respective cluster pattern (Supplementary Methods Figure 1). This resulted in generation of cluster patterns with 10, 9, 6 and 5 clusters respectively (Figure 2 and Supplementary Methods Table 1).


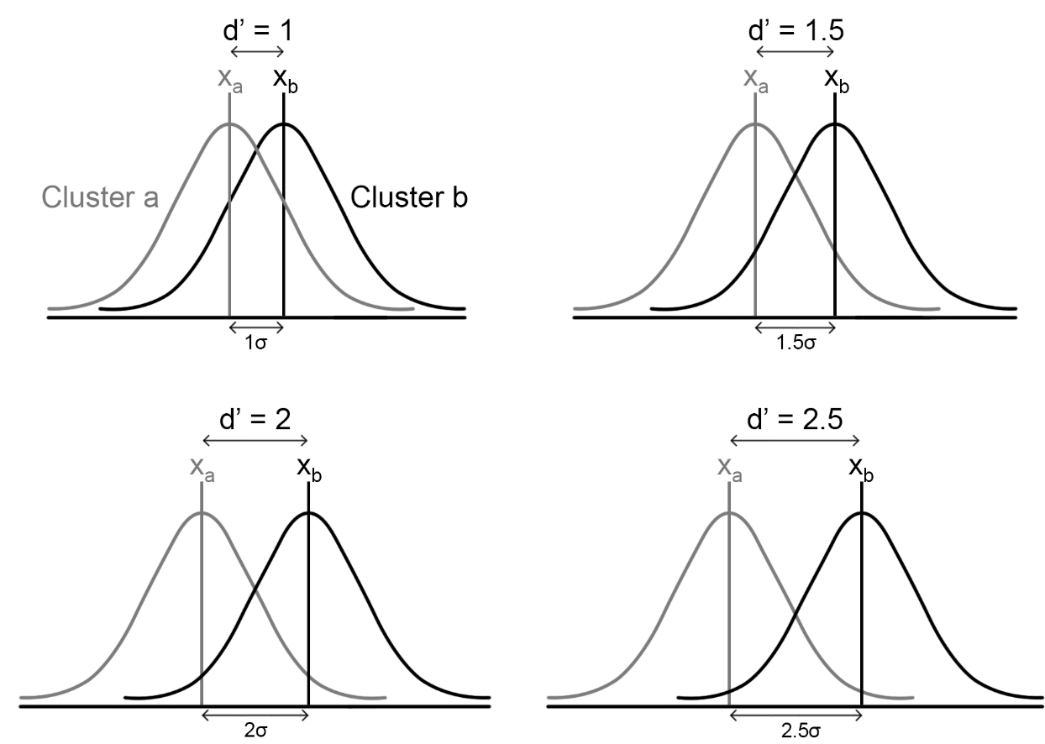


***Supplementary Methods Figure 1.*** *Schematic describing d’ statistic calculations to determine cluster separability. For the criterion of d’ >1 between clusters, for each cluster comparison a minimum of 1 unit of standard deviation (σ) was required to separate cluster means (x­_a_ and x_b_). This process was repeated for criteria of minimum 1.5σ separation (d’>1.5), 2σ separation (d’>2) and 2.5σ separation (d’>2.5) to generate four cluster patterns in total.*

***Supplementary Methods Table 1.*** *Ganglion cell-inner plexiform layer (GCIPL) thicknesses in microns for each cluster within each cluster pattern, derived from the normative model cohort prior to age-correction. Values are mean ± standard deviation GCIPL thicknesses.*

|  | **10-Cluster Pattern (1σ)** | **9-Cluster Pattern (1.5σ)** | **6-Cluster Pattern (2σ)** | **5-Cluster Pattern (2.5σ)** |
| --- | --- | --- | --- | --- |
| Cluster 1 | 99.0 ± 4.0 | 99.0 ± 4.0 | 99.0 ± 4.0 | 99.0 ± 4.0 |
| Cluster 2 | 90.7 ± 3.9 | 86.3 ± 4.9 | 83.8 ± 5.6 | 83.8 ± 5.6 |
| Cluster 3 | 84.9 ± 4.4 | 79.3 ± 3.4 | 69.9 ± 4.6 | 65.6 ± 6.1 |
| Cluster 4 | 79.3 ± 3.4 | 72.6 ± 4.0 | 60.9 ± 3.4 | 52.3 ± 3.8 |
| Cluster 5 | 72.6 ± 4.0 | 67.1 ± 3.3 | 52.3 ± 3.8 | 41.4 ± 3.4 |
| Cluster 6 | 67.1 ± 3.3 | 60.9 ± 3.4 | 41.4 ± 3.4 |  |
| Cluster 7 | 60.9 ± 3.4 | 54.8 ± 3.0 |  |  |
| Cluster 8 | 54.8 ± 3.0 | 50.2 ± 3.0 |  |  |
| Cluster 9 | 50.2 ± 3.0 | 41.4 ± 3.4 |  |  |
| Cluster 10 | 41.4 ± 3.4 |  |  |  |

*σ, standard deviations*

In locations corresponding to the same VF test location and variable cluster assignment depending on fovea to optic disc tilt (Supplementary Methods Figure 2A), fovea to optic disc tilt cut-offs determining cluster assignment were derived. For each location demonstrating variable cluster assignment, mean GCIPL thickness was calculated across all participants allocated to the same fovea to optic disc tilt, and the midline between the minimum of the cluster with thicker GCIPL measurements and the maximum of the cluster with thinner GCIPL measurements was calculated. GCIPL thickness was plotted as a function of fovea to optic disc tilt, and the best fit polynomial regression model was applied based on extra sums-of-squares F-test comparisons between quadratic, cubic and quartic regression models for each location (Supplementary Methods Figure 2B). The intersection between the regression model and midline derived above was subsequently utilized as the fovea to optic disc tilt cut-off determining cluster assignment (Supplementary Methods Table 2). In cases where the regression model and midline did not intersect, the cluster at that location for the fovea to optic disc tilt in question was reassigned to match the cluster assignment suggested by the regression model.


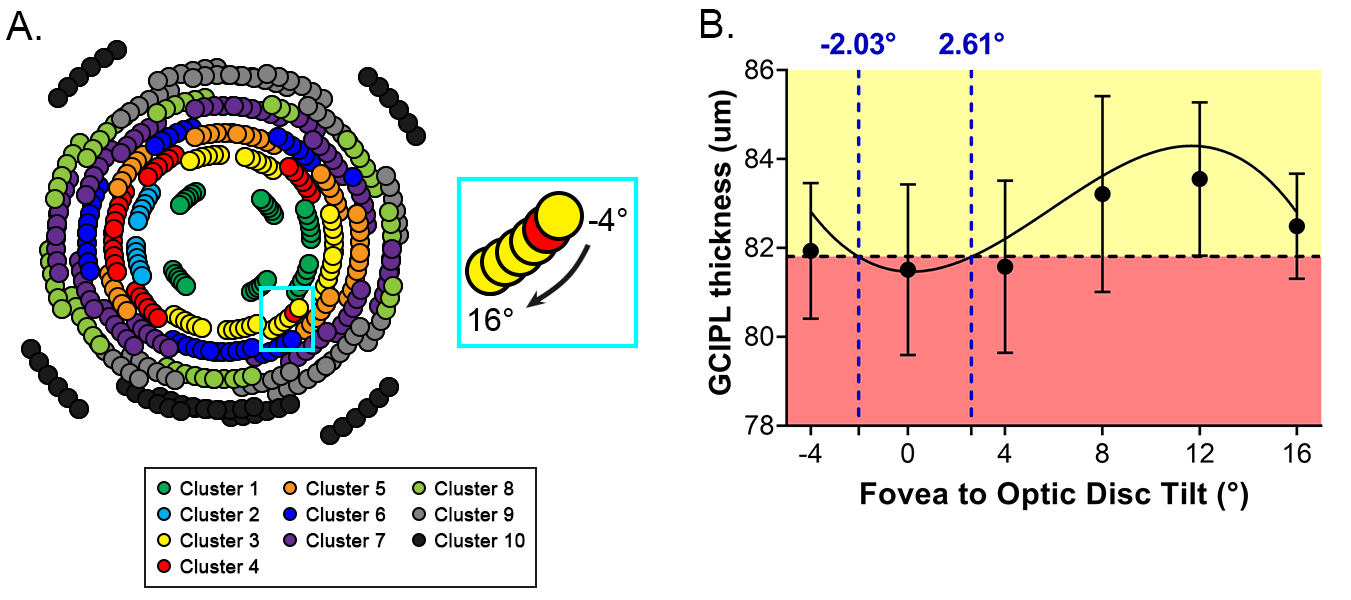


***Supplementary Methods Figure 2****. Schematic describing cluster assignment procedure in test locations with variable cluster assignment at different fovea to optic disc tilts. A. An example of a location demonstrating variable cluster assignment at different fovea to optic disc tilts within the 10-cluster pattern; at 0° the location has been assigned to Cluster 4, while at all other fovea to optic disc tilts it has been assigned to Cluster 3. B. Mean GCIPL thickness plotted as a function of fovea to optic disc tilt best followed a cubic regression model. The horizontal black dashed line indicates the midline between the minimum mean GCIPL thickness in Cluster 3 (shaded yellow background) and the maximum mean GCIPL thickness in Cluster 4 (shaded red background). The intersections between the midline and regression model at -2.03° and 2.61° (blue dashed lines) were used as the fovea to optic disc tilt cut-offs determining assignment to Clusters 3 or 4.*

***Supplementary Methods Table 2.*** *Fovea to optic disc tilt cut-offs determining cluster assignment in cluster patterns derived from hierarchical algorithms, at test grid locations where variable cluster assignment occurred. Labelling of clusters and locations are as per Figure 2 and Supplementary Methods Figure 3 respectively.*

| **Location** | **Fovea to Optic Disc Cut-Off (°)** | **Cluster Assignments** | | **Location** | **Fovea to Optic Disc Cut-Off (°)** | **Cluster Assignments** | |
| --- | --- | --- | --- | --- | --- | --- | --- |
|  |  | **< Cut-Off** | **> Cut-Off** |  |  | **< Cut-Off** | **> Cut-Off** |
| **10-Cluster Pattern (1σ)** | | | | **9-Cluster Pattern (1.5σ)** | | | |
| 11 | 5.89 | Cluster 8 | Cluster 7 | 11 | 5.89 | Cluster 7 | Cluster 6 |
| 29 | -1.91 | Cluster 6 | Cluster 7 | 29 | -1.91 | Cluster 5 | Cluster 6 |
| 57 | -2.03 | Cluster 3 | Cluster 4 | 57 | -2.03 | Cluster 2 | Cluster 3 |
|  | 2.61 | Cluster 4 | Cluster 3 |  | 2.61 | Cluster 3 | Cluster 2 |
| 60 | 4.92 | Cluster 8 | Cluster 9 | 60 | 4.92 | Cluster 7 | Cluster 8 |
| 66 | 14.42 | Cluster 6 | Cluster 7 | 66 | 14.42 | Cluster 5 | Cluster 6 |
| **6-Cluster Pattern (2σ)** | | | | **5-Cluster Pattern (2.5σ)** | | | |
| 11 | 5.89 | Cluster 5 | Cluster 4 | 11 | 5.89 | Cluster 4 | Cluster 3 |
| 29 | -1.91 | Cluster 3 | Cluster 4 |  |  |  |  |
| 66 | 14.42 | Cluster 3 | Cluster 4 |  |  |  |  |

σ, standard deviations


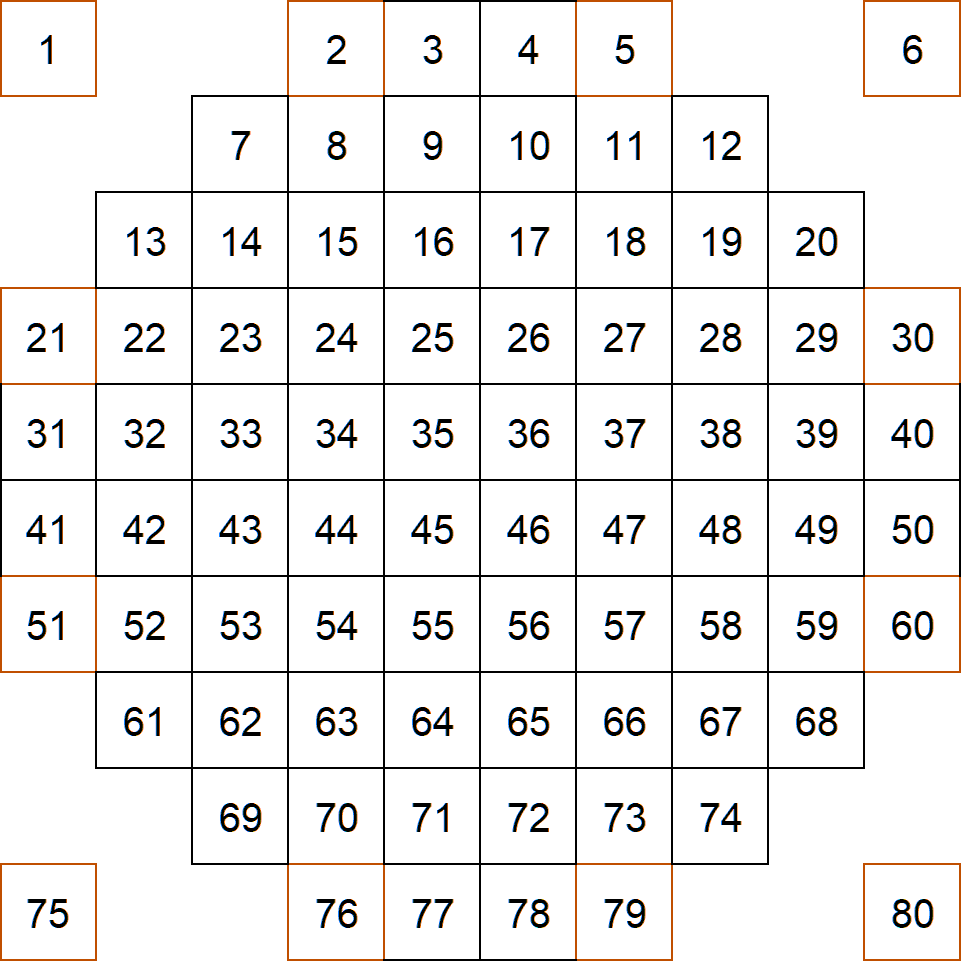


***Supplementary Methods Figure 3.*** *Labelling of test grid locations extracted using the MATLAB algorithm. Locations with black borders indicate those from the Humphrey Field Analyzer (HFA) 10-2 test grid, while locations with orange borders indicate paracentral locations within the HFA 30-2 test grid.*

**3. Age-Regression Models**

Regression models of ageing based on the generated cluster patterns were derived to enable identification of age-correction factors, to subsequently be applied to the clustered normative models to minimize variability due to normal aging changes.^3, 4^ As per previously described methods,^3^ for each cluster pattern mean GCIPL measurements were calculated per cluster and per decade bracket, and extra sums-of-squares F-tests were used to compare quadratic and linear regression models applied to describe GCIPL thickness as a function of age. Extra sums-of-squares F-tests were also applied to compare regression models between clusters within individual cluster patterns, with non-significant differences between clusters conferring the application of the same regression coefficients to these clusters. Age-correction factors were derived from the models demonstrating the best fit to the data (Supplementary Methods Table 3).

***Supplementary Methods Table 3.*** *Cluster-based aging regression models of the GCIPL derived from hierarchical cluster analysis applied to the normative model cohort. Quadratic regression models are described by the equation* $y=ax^{2}+bx+c$ *while linear regression models are described by* $y=bx+c$*, where y represents the age-corrected GCIPL thickness and x represents the original GCIPL thickness. The coefficients of determination (R^2^) values are derived from the regression models generated from the healthy model cohort. For subsequent age correction, the constant (c) is derived for individual participants.*

|  | **c** | **b** | **a** | **R^2^** |
| --- | --- | --- | --- | --- |
| **10-Cluster Pattern (1σ)** |  |  |  |  |
| Cluster 1 | 97.5 | 0.27 | -0.004 | 0.97 |
| Cluster 2 | 89.2 | 0.27 | -0.004 | 0.94 |
| Cluster 3 | 83.7 | 0.27 | -0.004 | 0.95 |
| Cluster 4 | 78.1 | 0.27 | -0.004 | 0.97 |
| Cluster 5 | 71.1 | 0.27 | -0.004 | 0.99 |
| Cluster 6 | 65.7 | 0.27 | -0.004 | 0.93 |
| Cluster 7 | 65.5 | -0.09 |  | 0.83 |
| Cluster 8 | 59.4 | -0.09 |  | 0.84 |
| Cluster 9 | 54.9 | -0.09 |  | 0.89 |
| Cluster 10 | 46.0 | -0.09 |  | 0.29 |
| **9-Cluster Pattern (1.5σ)** |  |  |  |  |
| Cluster 1 | 97.9 | 0.25 | -0.004 | 0.97 |
| Cluster 2 | 85.3 | 0.25 | -0.004 | 0.97 |
| Cluster 3 | 78.5 | 0.25 | -0.004 | 0.97 |
| Cluster 4 | 71.5 | 0.25 | -0.004 | 0.99 |
| Cluster 5 | 66.1 | 0.25 | -0.004 | 0.93 |
| Cluster 6 | 65.5 | -0.09 |  | 0.83 |
| Cluster 7 | 59.4 | -0.09 |  | 0.84 |
| Cluster 8 | 54.9 | -0.09 |  | 0.89 |
| Cluster 9 | 46.0 | -0.09 |  | 0.29 |
| **6-Cluster Pattern (2σ)** |  |  |  |  |
| Cluster 1 | 97.2 | 0.26 | -0.004 | 0.96 |
| Cluster 2 | 80.9 | 0.26 | -0.004 | 0.97 |
| Cluster 3 | 73.0 | 0.26 | -0.004 | 0.97 |
| Cluster 4 | 59.2 | 0.26 | -0.004 | 0.90 |
| Cluster 5 | 56.1 | -0.08 |  | 0.82 |
| Cluster 6 | 45.2 | -0.08 |  | 0.61 |
| **5-Cluster Pattern (2.5σ)** |  |  |  |  |
| Cluster 1 | 97.5 | 0.26 | -0.004 | 0.97 |
| Cluster 2 | 82.3 | 0.26 | -0.004 | 0.99 |
| Cluster 3 | 64.1 | 0.26 | -0.004 | 0.96 |
| Cluster 4 | 56.1 | -0.08 |  | 0.82 |
| Cluster 5 | 45.2 | -0.08 |  | 0.61 |

*c, constant; b, linear regression coefficient; a, quadratic regression coefficient; R^2^, coefficient of determination; σ, standard deviations*

**4. Refractive error analyses**

The final component to be considered in normative model development was refractive error. After age-correction and pooling as per the derived cluster patterns, Spearman correlations between refractive error and GCIPL thickness were computed. While the data followed a normal distribution, Spearman correlations were chosen over Pearson’s correlations to negate assumptions of a linear relationship between these variables. Across all clusters and cluster patterns, significant Spearman correlations were observed at the peripheral-most clusters and in one mid-peripheral cluster in the 9-Cluster Pattern (Supplementary Methods Table 4); given the minimal influence of refractive error on the majority of extracted GCIPL locations, refractive error was not incorporated into the normative models.

***Supplementary Methods Table 4.*** *Spearman correlations (r) between refractive error of the healthy model cohort and cluster-based ganglion cell-inner plexiform layer (GCIPL) thickness for cluster patterns derived from hierarchical cluster analysis.*

|  | **r** | **P value** |
| --- | --- | --- |
| **10-Cluster Pattern (1σ)** |  |  |
| Cluster 1 | -0.005 | 0.92 |
| Cluster 2 | -0.020 | 0.65 |
| Cluster 3 | -0.058 | 0.20 |
| Cluster 4 | -0.022 | 0.62 |
| Cluster 5 | -0.048 | 0.29 |
| Cluster 6 | 0.019 | 0.68 |
| Cluster 7 | -0.010 | 0.82 |
| Cluster 8 | 0.009 | 0.84 |
| Cluster 9 | 0.003 | 0.94 |
| Cluster 10 | 0.122 | 0.01 |
| **9-Cluster Pattern (1.5σ)** |  |  |
| Cluster 1 | 0.06 | 0.60 |
| Cluster 2 | -0.23 | 0.06 |
| Cluster 3 | -0.01 | 0.93 |
| Cluster 4 | -0.24 | 0.04 |
| Cluster 5 | -0.09 | 0.46 |
| Cluster 6 | -0.14 | 0.23 |
| Cluster 7 | -0.08 | 0.51 |
| Cluster 8 | -0.02 | 0.88 |
| Cluster 9 | 0.36 | 0.002 |
| **6-Cluster Pattern (2σ)** |  |  |
| Cluster 1 | -0.02 | 0.71 |
| Cluster 2 | -0.06 | 0.21 |
| Cluster 3 | -0.03 | 0.50 |
| Cluster 4 | 0.04 | 0.37 |
| Cluster 5 | -0.01 | 0.76 |
| Cluster 6 | 0.09 | 0.04 |
| **5-Cluster Pattern (2.5σ)** |  |  |
| Cluster 1 | -0.01 | 0.84 |
| Cluster 2 | -0.05 | 0.27 |
| Cluster 3 | 0.005 | 0.92 |
| Cluster 4 | -0.01 | 0.76 |
| Cluster 5 | 0.09 | 0.04 |

*σ, standard deviations*

**References for Supplementary Methods**

1. Tong J, Alonso-Caneiro D, Kalloniatis M, Zangerl B. Custom extraction of macular ganglion cell-inner plexiform layer thickness more precisely colocalizes structural measurements with visual fields test grids. *Sci Rep* 2020;10(1):18527.

2. Phu J, Khuu SK, Nivison-Smith L, et al. Pattern recognition analysis reveals unique contrast sensitivity isocontours using static perimetry thresholds across the visual field. *Invest Ophthalmol Vis Sci* 2017;58(11):4863-76.

3. Tong J, Phu J, Khuu SK, et al. Development of a spatial model of age-related change in the macular ganglion cell layer to predict function from structural changes. *Am J Ophthalmol* 2019;208:166-77.

4. Yoshioka N, Zangerl B, Nivison-Smith L, et al. Pattern recognition analysis of age-related retinal ganglion cell signatures in the human eye. *Invest Ophthalmol Vis Sci* 2017;58(7):3086-99.


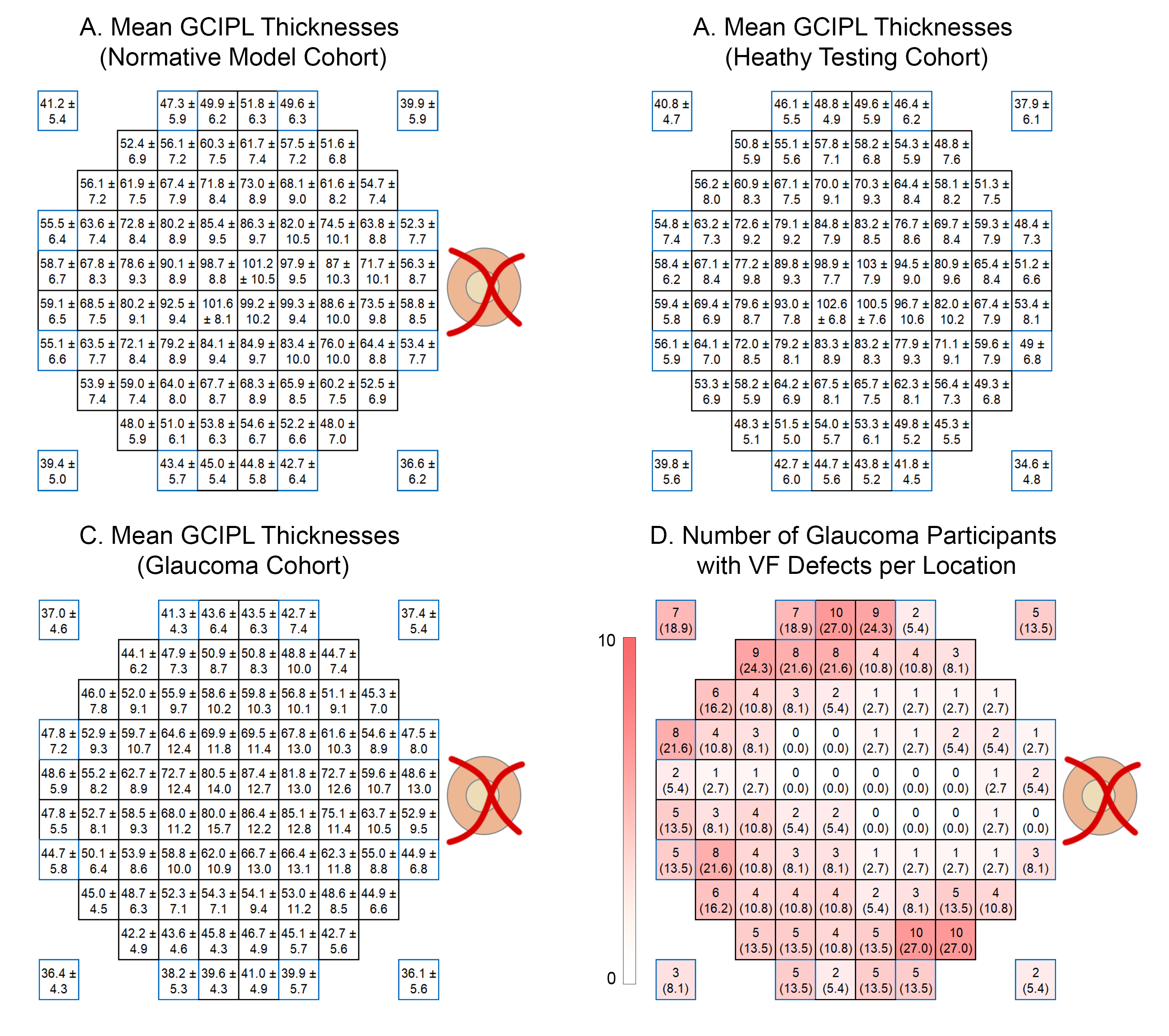


**Supplementary Figure 1.** Mean and standard deviation ganglion cell-inner plexiform layer (GCIPL) thickness values as extracted using the MATLAB algorithm at 0° grid tilt from the A. normative model cohort, B. healthy testing cohort and C. the glaucoma cohort, with all values converted to right eye format. Locations highlighted in blue indicate the paracentral locations in the 30-2 Humphrey Field Analyzer (HFA) test grid, while locations in black indicate those from the 10-2 HFA test grid. D. Number of participants within the glaucoma cohort with visual field (VF) locations classified as VF defective across all tested locations. Numbers in brackets indicate percentage of the glaucoma cohort with VF defective classifications for each location.


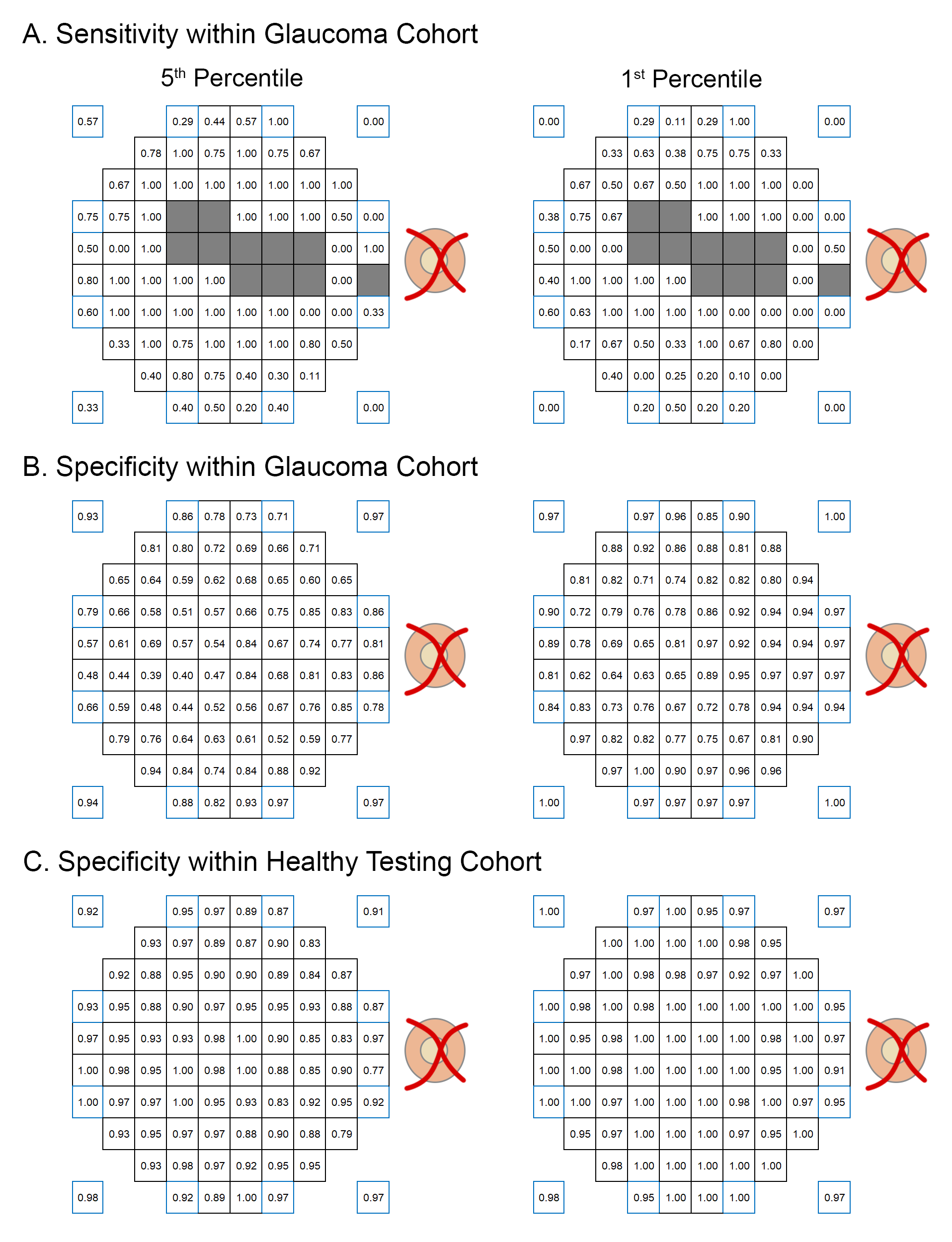


**Supplementary Figure 2.** Pointwise A. sensitivities and B. specificities within the glaucoma cohort, and C. specificities within the healthy testing cohort, across the macula using 5^th^ (right column) and 1^st^ percentile limits (left column) generated from the normative model cohort. Locations with black borders indicate those from the Humphrey Field Analyzer (HFA) 10-2 test grid, while locations with blue borders indicate paracentral locations within the HFA 30-2 test grid. Locations shaded in grey are those where no glaucoma participants demonstrated a VF defect, and therefore sensitivities could not be derived.

**Supplementary Table 1.** Cluster-specific sensitivities and specificities of 5^th^ and 1^st^ percentile limit ganglion cell-inner plexiform layer (GCIPL) cut-offs in identifying co-localized visual field (VF)-defective and VF-normal results in the glaucoma and healthy cohorts.

|  | **5^th^ Percentile** | | | **1^st^ Percentile** | | |
| --- | --- | --- | --- | --- | --- | --- |
|  | **Glaucoma** | | **Healthy** | **Glaucoma** | | **Healthy** |
|  | **Sensitivity** | **Specificity** | **Specificity** | **Sensitivity** | **Specificity** | **Specificity** |
| **10-Cluster Pattern (1σ)** | | | | | | |
| Cluster 1 | 1.00 | 0.72 | 0.92 | 1.00 | 0.88 | 0.98 |
| Cluster 2 | 1.00 | 0.56 | 0.94 | 1.00 | 0.67 | 1.00 |
| Cluster 3 | 0.83 | 0.69 | 0.94 | 0.83 | 0.83 | 0.99 |
| Cluster 4 | 1.00 | 0.66 | 0.90 | 0.89 | 0.79 | 0.98 |
| Cluster 5 | 0.80 | 0.79 | 0.93 | 0.47 | 0.90 | 0.98 |
| Cluster 6 | 0.83 | 0.68 | 0.87 | 0.44 | 0.85 | 0.96 |
| Cluster 7 | 0.73 | 0.73 | 0.90 | 0.54 | 0.89 | 0.95 |
| Cluster 8 | 0.62 | 0.81 | 0.91 | 0.28 | 0.94 | 0.98 |
| Cluster 9 | 0.25 | 0.90 | 0.93 | 0.08 | 0.97 | 0.98 |
| Cluster 10 | 0.19 | 0.97 | 0.92 | 0.03 | 1.00 | 0.98 |
| **9-Cluster Pattern (1.5σ)** | | | | | | |
| Cluster 1 | 1.00 | 0.72 | 0.95 | 1.00 | 0.88 | 1.00 |
| Cluster 2 | 1.00 | 0.66 | 0.89 | 0.88 | 0.82 | 0.98 |
| Cluster 3 | 1.00 | 0.66 | 0.92 | 0.89 | 0.79 | 0.98 |
| Cluster 4 | 0.80 | 0.79 | 0.88 | 0.47 | 0.90 | 0.96 |
| Cluster 5 | 0.83 | 0.68 | 0.90 | 0.44 | 0.85 | 0.95 |
| Cluster 6 | 0.73 | 0.73 | 0.91 | 0.54 | 0.89 | 0.98 |
| Cluster 7 | 0.62 | 0.81 | 0.93 | 0.28 | 0.94 | 0.98 |
| Cluster 8 | 0.25 | 0.90 | 0.92 | 0.08 | 0.97 | 0.98 |
| Cluster 9 | 0.19 | 0.97 | 0.95 | 0.03 | 1.00 | 0.99 |
| **6-Cluster Pattern (2σ)** | | | | | | |
| Cluster 1 | 1.00 | 0.71 | 0.95 | 1.00 | 0.88 | 1.00 |
| Cluster 2 | 0.94 | 0.68 | 0.90 | 0.82 | 0.80 | 0.98 |
| Cluster 3 | 0.73 | 0.73 | 0.90 | 0.45 | 0.87 | 0.97 |
| Cluster 4 | 0.71 | 0.77 | 0.90 | 0.46 | 0.91 | 0.98 |
| Cluster 5 | 0.39 | 0.86 | 0.92 | 0.13 | 0.97 | 0.98 |
| Cluster 6 | 0.22 | 0.96 | 0.95 | 0.03 | 1.00 | 0.99 |
| **5-Cluster Pattern (2.5σ)** | | | | | | |
| Cluster 1 | 1.00 | 0.72 | 0.95 | 1.00 | 0.88 | 1.00 |
| Cluster 2 | 0.94 | 0.68 | 0.90 | 0.82 | 0.80 | 0.98 |
| Cluster 3 | 0.66 | 0.79 | 0.91 | 0.47 | 0.91 | 0.97 |
| Cluster 4 | 0.39 | 0.86 | 0.92 | 0.13 | 0.97 | 0.98 |
| Cluster 5 | 0.22 | 0.96 | 0.95 | 0.03 | 1.00 | 0.99 |

σ, standard deviations**Supplementary Table 2**. Accuracy of ganglion cell-inner plexiform layer (GCIPL) cut-offs based from the maximum Youden’s indices in identifying co-localized visual field (VF)-defective and VF-normal results in the glaucoma and healthy testing cohorts. Youden’s indices were calculated from receiver operator characteristic (ROC) curves in from the glaucoma cohort. 95% confidence intervals are shown in brackets.

|  | **Glaucoma** | | **Healthy** |
| --- | --- | --- | --- |
|  | **Sensitivity** | **Specificity** | **Specificity** |
| **10-Cluster Pattern (1σ)** | 0.69 (0.63-0.75) | 0.81 (0.79-0.82) | 0.90 (0.89-0.92) |
| **9-Cluster Pattern (1.5σ)** | 0.69 (0.63-0.75) | 0.80 (0.79-0.82) | 0.90 (0.89-0.92) |
| **6-Cluster Pattern (2σ)** | 0.71 (0.66-0.77) | 0.77 (0.75-0.78) | 0.88 (0.87-0.89) |
| **5-Cluster Pattern (2.5σ)** | 0.72 (0.67-0.78) | 0.75 (0.74-0.77) | 0.87 (0.86-0.88) |

σ, standard deviations

**Supplementary Table 3.** Cluster-specific sensitivities and specificities of ganglion cell-inner plexiform layer (GCIPL) cut-offs based from the maximum Youden’s indices in identifying co-localized visual field (VF)-defective and VF-normal results in the glaucoma and healthy cohorts.

|  | **Glaucoma** | | **Healthy** |
| --- | --- | --- | --- |
|  | **Sensitivity** | **Specificity** | **Specificity** |
| **10-Cluster Pattern (1σ)** |  |  |  |
| Cluster 1 | 1.00 | 0.99 | 1.00 |
| Cluster 2 | 1.00 | 0.81 | 1.00 |
| Cluster 3 | 0.83 | 0.89 | 0.99 |
| Cluster 4 | 0.89 | 0.84 | 0.99 |
| Cluster 5 | 0.80 | 0.81 | 0.90 |
| Cluster 6 | 0.83 | 0.72 | 0.91 |
| Cluster 7 | 0.71 | 0.79 | 0.93 |
| Cluster 8 | 0.70 | 0.80 | 0.93 |
| Cluster 9 | 0.64 | 0.70 | 0.79 |
| Cluster 10 | 0.50 | 0.84 | 0.79 |
| **9-Cluster Pattern (1.5σ)** |  |  |  |
| Cluster 1 | 1.00 | 0.99 | 1.00 |
| Cluster 2 | 0.88 | 0.83 | 0.99 |
| Cluster 3 | 0.89 | 0.84 | 0.99 |
| Cluster 4 | 0.80 | 0.81 | 0.90 |
| Cluster 5 | 0.83 | 0.72 | 0.91 |
| Cluster 6 | 0.71 | 0.79 | 0.93 |
| Cluster 7 | 0.70 | 0.80 | 0.93 |
| Cluster 8 | 0.64 | 0.70 | 0.79 |
| Cluster 9 | 0.50 | 0.84 | 0.79 |
| **6-Cluster Pattern (2σ)** |  |  |  |
| Cluster 1 | 1.00 | 0.99 | 1.00 |
| Cluster 2 | 0.94 | 0.76 | 0.96 |
| Cluster 3 | 0.88 | 0.65 | 0.85 |
| Cluster 4 | 0.69 | 0.82 | 0.92 |
| Cluster 5 | 0.70 | 0.70 | 0.82 |
| Cluster 6 | 0.50 | 0.84 | 0.80 |
| **5-Cluster Pattern (2.5σ)** |  |  |  |
| Cluster 1 | 1.00 | 0.99 | 1.00 |
| Cluster 2 | 0.94 | 0.76 | 0.96 |
| Cluster 3 | 0.80 | 0.71 | 0.85 |
| Cluster 4 | 0.70 | 0.70 | 0.82 |
| Cluster 5 | 0.50 | 0.84 | 0.80 |

σ, standard deviations
